# 

**Published:** 1919-08-23

**Authors:** 


### Limit of Health Insurance.

By the National Health Insurance Act, 1919, which has just received the Royal Assent, the limit of remuneration up to which persons employed otherwise than by way of manual labour are liable to compulsory health insurance has been raised from £160 to £250 a year. Employers are accordingly required for the future to pay health insurance contributions in respect of such persons. Within a limited period and under certain conditions a certificate of exemption may be claimed by the worker, and on the grant of such a certificate the employer's contributions (3d. a week) are alone payable. Forms of application for exemption will be obtainable shortly at any Post Office.

